# Randomized Comparison of Nylon Versus Absorbing Polyglactin 910 for Fascial Closure in Caesarean Section

**DOI:** 10.5812/ircmj.12580

**Published:** 2014-04-05

**Authors:** Kolsoum Rezaie Kahkhaie, Khadije Rezaie Keikhaie, Aziz Shahreki Vahed, Mahboobeh Shirazi, Nooshin Amjadi

**Affiliations:** 1 Medicinal Plants Research Center, Zabol University of Medical Sciences (ZBUMS), Zabol, IR Iran; 2 Department of Perinatalogy, Zabol University of Medical Sciences (ZBUMS), Zabol, IR Iran; 3 Nursing and Midwifery School, Zabol University of Medical Sciences (ZBUMS), Zabol, IR Iran; 4 Maternal, Fetal and Neonatal Research Center, Tehran University of Medical Sciences, IR Iran

**Keywords:** Surgery, Sutures, Caesarean Section, Polygalactin 910

## Abstract

**Background:**

Regardless of numerous advances in surgical techniques, selection of the best technique to sew up wounds and the best suture material are still controversial. Several postoperative complications, including wound infection, stitched wound, chronic incision pain, wound dehiscence and hernia stitches result from many factors such as used suture material.

**Objectives:**

The aim of the present study was to investigate the complications of pfannenstiel incision and nylon/ polyglactin 910 sutures utilization in patients undergoing c-section cesarean.

**Patients and Methods:**

This clinical trial was conducted on 120 women who underwent caesarean section at Imam-Ali hospital in Zabol, Iran. In this study, patients were equally divided into two groups of 60 people (50% in nylon suture and 50% in polyglactin 910 sutures). Patients of the two groups were investigated by a gynecologist 24-48 hours after the operation, a week later and on the sixth month of surgery. Moreover, time of wound dehiscence and treatment duration, the level of sinus infection, chronic incision pain and incision hernia were studied. The results were analyzed by SPSS software. P ≤ 0.05 was considered as statistically signiﬁcant.

**Results:**

One hundred and twenty patients undergoing a cesarean section at Imam-Ali hospital in Zabol were recruited into the study, 60 in the Nylon group and 60 in Polyglactin 910group. Our data demonstrated a statistically higher incidence of suture sinus and chronic incision pain in the nylon group (P < 0.05). No statistically signiﬁcant difference in wound stitch and incision hernia was demonstrated between the suture groups.

**Conclusions:**

The results of our trial did not demonstrate a significant difference between absorbing polyglactin 910 (PDS) and nylon regarding incision hernia, wound infection and wound dehiscence. However, subjects sutured with PDS were less likely to experience chronic incision pain and wound stitch. Therefore, PDS appears to be the optimal choice for fascial closure after cesarean section.

## 1. Background

Regardless of numerous advances in surgical techniques, selection of the best technique to sew up wounds and the best used suture material are still controversial. Several postoperative complications, including wound infection, stitched wound, chronic incision pain, wound dehiscence and hernia stitches result from many factors such as the suture material used (1, 2). Using sutures for wound closure and bleeding control backs to four thousand years ago (3). A reliable capability of absorption during the healing period and no sensitivity are among the most important characteristics of an ideal suture (4).The level of tissue reaction to the suture material is one of the most significant features in choosing among various types of suture materials used to wound closure (5). Caesarean delivery is the most common major surgical procedure for women worldwide (6). Caesarean section is a major surgery along with an incision in skin of the abdominal wall muscles and the uterus wall. Those women undergone a c-section complain about abdominal and incision pains (7). Incision repair following abdominal surgery is of great importance. Disregarding the aforementioned subject could result in different complications such as the risk of wound dehiscence, chronic incision pain, sinus infection and incision hernia leading to hospital readmissions and waste of time and money (8-10). Surgical technique and the type of suture are the only factors directly controlled by the surgeon and playing a crucial role in wound healing (9, 11). Different types of sutures, including absorbable /non absorbable, natural /synthetic and monofilament /multifilament have their advantages as well as disadvantages in wound repair (12). Nylon, a non-absorbable synthetic suture, is extracted from acid hexamethylene adipic acid. Nylon suture composed of mono or multi filament nylon yarns, has moderate tensile strength and produced the mildest tissue reaction (3, 4, 13). Polyglactin 910, a synthetic absorbable suture, is completely absorbed in 15 days (14). Greenberg et al. conducted a randomized trial on 1361 women underwent cesarean section to evaluate the healing characteristics of chromic versus fast-absorbing polyglactin 910. Their finding demonstrated that there was a significant reduction in uterine cramping pain in subjects assigned to fast-absorbing polyglactin. Moreover, there was signiﬁcantly less scar pain experienced in the PDS group (14). A recent analysis of perineal repairs via polyglycolic suture and chromic catgut also reported that polyglycolic was a biologically probable suture with a lower local inflammatory reaction but its absorption takes longer. Catgut is rapidly absorbed but results in a local inflammatory reaction (15). Several studies were performed to find a model suture used in incision closure. Nevertheless, the ideal suture for closing abdominal fascia has not been determined yet. Consequently, the aim of the present study was to investigate the complications of pfannenstiel incision repair and nylon/ polyglactin 910 sutures utilization in patients undergoing c-section cesarean. Obviously, any of the aforesaid methods of suturing with dramatically approved preferences could be employed as a procedure to prevent and reduce the incidence of postoperative complications of caesarean section.

## 2. Objectives

The aim of the present study was to investigate the complications of pfannenstiel incision repair and nylon/ polyglactin 910 sutures utilization in patients undergoing c-section cesarean.

## 3. Patients and Methods

This clinical trial was conducted on 120 women who underwent caesarean section at Imam-Ali hospital in Zabol, Iran. The study was initiated following approval from the University Research & Ethics Committee. In this study, patients were equally divided into two groups of 60 participants (50% in nylon suture and 50% in polyglactin 910 sutures). Prior to caesarean sections, some information was provided regarding the research goals, type of surgery, utilization of sutures and voluntary participation of patients in the project by a gynecologist (and obstetrician). Informed consent was obtained from patients willing to participate in this study. Patients' dissatisfaction, history of pre-existing conditions, including diabetes, hypertension and cardiovascular disorders as well as anemia, previous cesarean delivery, infection, malignancy and utilization of corticosteroids drugs were considered prior to the investigation. Therefore, history taking such as malignant diseases, physical examination, a complete blood count (CBC), blood glucose testing, and urea and creatinine levels were performed for patients participated in this study. Variables including age, party, gestational age and the reason of caesarean section were also recorded. All caesarean deliveries were operated by obstetricians/ gynecologists in the hospital. Using the same procedure, Polyglactin 910 sutures (No. 1, Supa Factory, Approved by the Ministry of Health and Medical Education of Iran) and nylon sutures (No. 1, Supa Factory) were used during cesarean section in cases and controls, respectively. A 7-14 cm-long incision was made in all patients and the same continuous suture technique was performed in the both groups. All patients were well matched regarding age, body mass index (BMI) and cut lengths of suture. A gynecologist evaluated patients of the two groups 24-48 hours after the operation, a week later and thereafter on the sixth month postoperatively. Moreover, time of wound dehiscence and treatment duration, the level of sinus infection, chronic incision pain and incision hernia were studied. Chronic incision pain was associated with non-healing wounds six weeks postoperatively. Suture sinus was reported when a sinus was present in the suture tract (16). The results were analyzed by using SPSS software. P ≤ 0.05 was considered as statistically signiﬁcant.

## 4. Results

One hundred and twenty patients undergoing a cesarean section at Imam-Ali hospital in Zabol were recruited into the study, 60 in the Nylon group and 60 in the polyglactin 910 group. The results showed that 60% of patients were older than 30 years (5/24+29/50), most surgeons sampled (69.2%) represented BMI below 22%. The level of blood glucose in 82.5% of patients was below 110, while it was higher than 110 in 17.5% of cases. Additionally, our data demonstrated a statistically higher incidence of suture sinus in nylon group (P < 0.05%), with eight patients, and no cases of the wound stitch in polyglactin group. Twelve patients in a nylon group experienced chronic incision pain, while there was only one such patient in the polyglactin group which was a statistically significant result. Wound infection was observed in 2 and 3 of specimens belonged to polyglactin and nylon groups, respectively. No statistically signiﬁcant difference in wound stitch and incision hernia was demonstrated between the suture groups.

## 5. Discussion

Comparison of complications of Polydioxanone (PDS) and Polyamide (Nylon) sutures in the closure of midline incision (Laparotomy), performed by Eshghi et al., represented a statistically signiﬁcantly lower incidence of the prevalence and severity of chronic pain after surgery and suture sinus formation in the study group compared to the control group. Some complications such as wound infection, wound dehiscence and incision hernia were not statistically different between the two suture groups (17). The results of the present study indicated that the severity of chronic pain in patients undergoing caesarean section with polyglactin 910 sutures was lower than those using nylon sutures. Our results are consistent with the aforementioned report. Consequently, two suture groups were not different regarding wound infection, incisional hernia and wound dehiscence, whereas PDS sutures resulted in wound stitch formation and reduction of chronic pain postoperatively. Therefore, PDS suture could be considered as a proper alternative for nylon suture in healing of surgical incisions (9). A definitive randomized controlled trial comparing fast-absorbing polyglactin 910 versus chromic sutures was conducted on 1361 patients. The results demonstrated that there was a significant reduction in uterine pain 24-48 hours postoperatively in patients randomly utilized fast-absorbing polyglactin 910 sutures (p= 0.006). In 10-14 days after surgery, there were no statistically significant differences between the two groups. Although, there was a meaningful reduction in uterine cramping pain 6-8 weeks after surgery (p= 0.17), also a significant reduction in utilization of analgesic drugs in specimens randomly assigned to fast-absorbing polyglactin 910 sutures. Ultimately, there was no difference in the rest of residual sutures and wound stitches 6-8 weeks after surgery (14).

Upton et al. assessed the effect of suture materials (a polyglycolic suture and chromic catgut) used in perineal repairs on 391 women. Patients with a live singleton birth at 34 week of gestational age or more, who was primipara or required perineal repair due to either an episiotomy or the first or second degree tear participated in this randomized controlled trial. The results of this study demonstrated that 106 of the 194 (55%) women allocated to polyglycolic suture were primipara compared with 79 (40%) of the 197 women allocated to catgut. Women sutured with polyglycolic were less likely to experience perineal pain for three days postpartum compared with women sutured with catgut, but by six months postpartum were somewhat more likely to experience perineal pain, dyspareunia and require removal of a suture. The results represented that reduced short-term perineal pain in women repaired with polyglycolic was similar to women allocated to polyglycolic suture catgut. Their finding illustrated that polyglycolic is biologically plausible and causes a lower local inflammatory reaction, but its absorption takes longer. Catgut causes a local inflammatory reaction but is rapidly absorbed (15). We showed that polyglactin 910 sutures cause less local inflammatory reaction than nylon suture.

A report entitled comparison of wound disruption between three methods, including closure of subcutaneous with Polyglycolic versus closure of subcutaneous with plain and non-closure after cesarean section was performed by Nasrollahi et al. The results indicated that the differences in the incidence of infection in three study groups were not statistically significant (P ≤ 0.05) (18). In the recent study, suture sinus and wound infection did not show a significant difference between the both groups.

Aali et al. compared the utilization of plain catgut and vicryl in subcutaneous fat tissue closure of cesarean section through a prospective, randomized trial in which 1000 cases of cesarean section were performed over four years. Closure of subcutaneous fat layer was accomplished with plain catgut in the first and vicryl in the second group. Results indicated a significant difference between plain and vicryl materials regarding drainage of purulent discharge from the wound in the absence of fever or need for admission in the hospital (seven cases in the plain group versus one case in the vicryl group). Hematoma was found in two patients of plain closure group, while no hematoma was occurred in the vicryl group. Therefore, hematoma formation did not differ significantly in the both groups. No case of wound infection occurred. Regarding the high rate of cesarean section in Iran and considering the lower incidence of wound complications in vicryl utilization, replacing this suture material with plain catgut for closure of subcutaneous fat layer is highly recommended (19).

The results of our trial did not demonstrate a significant difference between fast-absorbing polyglactin 910 (PDS) and nylon regarding incision hernia, wound infection and wound dehiscence. However, subjects sutured with PDS were less likely to experience chronic incision pain and wound stitch. Therefore, PDS appears to be the optimal method of fascial closure after cesarean section.
